# Where Are the Knowledge Gaps in Menopause Across a Population? A National Cross-Sectional Survey in Wales

**DOI:** 10.3390/ijerph22020287

**Published:** 2025-02-14

**Authors:** Catherine A. Sharp, Nicola Dennis, Gemma Hobson, Marysia Hamilton-Kirkwood, Karen Hughes

**Affiliations:** 1 Policy and International Health Directorate, World Health Organization Collaborating Centre on Investment for Health & Well-Being, Public Health Wales, Cardiff CF10 4BZ, UK; catherine.sharp@wales.nhs.uk (C.A.S.); karen.hughes18@wales.nhs.uk (K.H.); 2 Applied Health Research Department, University of Birmingham, Birmingham B15 2TT, UK; 3 Public Health Wales, Cardiff CF10 4BZ, UK; gemma.hobson@wales.nhs.uk; 4 Public Health, Cwm Taf Morgannwg University Health Board, Merthyr Tydfil CF48 1BZ, UK; marysia.hamilton-kirkwood@wales.nhs.uk; 5 School of Health Sciences, Bangor University, Wrexham LL13 7YP, UK

**Keywords:** menopause, population, demographics, women, attitudes, perceptions

## Abstract

Background: A knowledge gap exists on population understanding and perspectives of menopause. To better support females, it is important to understand different population groups’ perceived knowledge of and attitudes towards menopause. This study explored perceived knowledge of menopause, awareness of menopausal symptoms, perceived negative impacts of menopause on females’ lives, and attitudes towards menopause. Methods: A national cross-sectional survey (n = 1020) was conducted with residents in Wales aged 16+ years as part of a larger population panel using a multi-method approach (online, telephone, and face-to-face), stratified by NHS health board area, age, sex, deprivation, and ethnicity. Questions on menopause were developed by the research team. Results: Nearly a third (31.1%) of participants reported having low knowledge of menopause, with younger age groups and males reporting the lowest knowledge. Hot flushes were the most recognised menopause symptom (92.2%). The symptom with the greatest gap in awareness between age groups, sexes, and deprivation quintiles was problems with memory or concentration. Most participants perceived menopause to have a large negative impact on females’ mental health (76.1%), working life (69.7%) and physical health (69.6%). Females and 30–49-year-olds were more likely to perceive large negative impacts across all three domains. Most participants (77.8%) agreed that more public discussion is needed on menopause to increase understanding. The greatest support for increasing public discussion was found amongst females and people aged 30–49 years old. Conclusions: At a population level, perceived knowledge of menopause and associated symptoms and perceived impacts on females’ lives varied across socio-demographic groups. While males were less knowledgeable than females, most males perceived negative impacts on females’ lives and agreed with action to improve the menopausal environment. Increasing knowledge in those who experience menopause and in those who support those who experience menopause could help females have a more positive transition process.

## 1. Introduction

Menopause is a natural process that marks the end of a woman’s reproductive life, typically occurring between the ages of 45 and 55 years [1]. It is a biopsychosocial phenomenon through which all females transition. Globally, the female population is increasing and is set to be proportionate to males by 2050 [2]. This, along with an ageing population, means an anticipated increase in the number of females who will experience menopause as well as an increase in post-menopausal life expectancy.

The physical and mental health symptoms females experience during menopause vary widely, resulting in both positive and negative consequences. A systematic review found females who reported more negative menopausal attitudes reported experiencing an increased number of symptoms [3]. A UK study found females with menopause symptoms experienced an average of seven symptoms, with 51% reporting a negative impact on their sex life, 43% on their work life and 36% on their social life [4]. A negative menopause experience is also exacerbated by existing inequalities, with a review finding various social determinants of health being associated with health and quality of life during menopause [5]. Females’ overall experience of menopause may vary due to sociocultural views around reproduction and ageing [6]. For instance, in some cultures, menopause is viewed as an illness; in others, it is viewed as a biological process or as a milestone to be celebrated. This difference in perception influences people’s attitudes towards menopause [7]. Menopause has long been a taboo subject in many societies, and females have often been found to be reticent to discuss the topic [8].

In many countries, societal approaches and attitudes towards menopause are changing. For example, in the UK, public discourse around menopause has shifted over the last decade. Alongside increasing media debate [9] and visible celebrity role models [10], in 2022 the UK government established a Menopause Taskforce with the stated aims of increasing access to treatment and reducing stigma. However, most research conducted to inform understanding of attitudes towards and views of menopause has focused specifically on menopausal population groups, including perimenopausal females [11,12,13], menopausal females [14,15], and post-menopausal females [7]. A minority of studies have focused on males whose female partners were experiencing menopause (e.g., [16,17]). A systematic review reported that males have a low understanding of menopause symptoms and struggle to cope with changes in their spouses’ behaviour and physiology [18]. Where studies have sought to recruit a non-menopausal sample, samples have been predominantly female (e.g., 84% females in [8]). Increased understanding of the perceptions and attitudes of different population groups could help improve the experience for females.

The evidence gap on population understanding of menopause means little is known about how attitudes towards and perceptions of menopause vary by socio-demographic factors. There is well-established evidence demonstrating certain population groups are at greater risk of experiencing health inequalities across a range of different health topics (e.g., people living in high areas of deprivation, people from ethnic minority backgrounds), and so it is important to investigate how such factors impact the area of menopause. Additionally, while the menopause phase undoubtedly has a direct impact on females, it is not experienced in isolation and can therefore also indirectly impact others in their environments (e.g., partners, children, parents, colleagues, and friends). Encouraging open dialogues about menopause and helping different population groups to have a better understanding of menopause could reduce the stigma experienced by many women [19]. Understanding awareness and perspectives at a population level will help inform the development of interventions to improve the environment for females while they go through this transition period. Increasing social support and education have been identified as approaches which can yield positive experiences for females while experiencing menopause [8] and could also likely benefit those in a supportive role.

This study aimed to address the aforementioned evidence gap to provide a national population perspective on (i) perceived knowledge of menopause; (ii) awareness of menopausal symptoms; (iii) perceived negative impact of menopause on the lives of females; (iv) attitudes towards menopause; and (v) outcomes of participants’ socio-demographic characteristics (i.e., age, sex, deprivation, and ethnicity).

## 2. Materials and Methods

Data for this study were collected in the April 2023 survey wave of the Time to Talk Public Health (TTPH) panel, a nationally representative panel of residents aged 16+ years in Wales (UK). TTPH was established by Public Health Wales (the national public health institute in Wales, UK) in November 2022 to enable regular public engagement through surveys to inform public health policy and practice. The panel has an overall sample size of approximately 2500 residents, with a target sample of 1000 participants completing each survey wave. Inclusion criteria for panel membership are being a resident of Wales (limited to one individual per household), aged 16+ years and cognitively able to participate. A market research company (MRC) was procured to work with Public Health Wales to establish and maintain the panel and collect survey data. The first phase of panel development (from November 2022 to March 2023) focused on sample recruitment and piloting of the survey process to inform routine implementation from April 2023.

The development and sampling framework for establishing and implementing the panel is available online [20]. In summary, to recruit a nationally representative sample to the panel sample, stratified quota targets were set by NHS local health board area, age, sex, deprivation quintile (as determined by the Welsh Index of Multiple Deprivation; WIMD; [21]) and ethnicity, based on mid-2020 population estimates [22]. WIMD is the official measure of relative deprivation for small geographical areas in Wales, comprised of eight domains of deprivation (income, employment, health, education, access to services, housing, community safety, physical environment). Small areas are ranked by score from most deprived to least deprived.

Over-sampling of population groups who are often under-represented in national surveys (e.g., low response rates) was designed into the quotas for the panel sample to achieve the required sample sizes. Population groups which were over-sampled included younger groups, those living in more deprived quintiles and ethnic minority groups. A multi-method, multi-step approach to initial recruitment was used, including telephone, face-to-face, social media advertising, and dissemination through networks.

Data collection for the April 2023 survey wave involved all panel members being invited to complete the survey via their pre-selected method (online or telephone). Reminder emails and chase calls were also undertaken to fill demographic gaps. To increase representation of 16–29-year-olds, an additional 100 face-to-face interviews were commissioned along with additional targeted social media advertising. Data collection took place over a 4-week period.

### 2.1. Questionnaire

TTPH surveys cover a range of public health topics based on organisational need, with questions developed in consultation with topic leads, drawing on existing and validated survey questions where possible. The data in this study are drawn from the survey undertaken in April 2023, with demographic data drawn from participants’ recruitment questions (collected between November 2022 and April 2023, depending on the participant recruitment date).

The April 2023 survey included four question sets on menopause; all answers were self-reported. The questions included in this study, their response options and data categorisation for analysis are shown in Table S1. Firstly, participants were asked how knowledgeable, if at all, they would say they were about menopause (response options: not at all knowledgeable, not very knowledgeable, fairly knowledgeable, very knowledgeable). Responses were dichotomised to identify perceived low knowledge (not at all or not very knowledgeable = 1, other responses = 0). Secondly, participants were asked to identify which of a list of eight menopausal symptoms they knew could be a symptom of menopause (hot flushes, difficulty sleeping, weight gain, changes to mood, headaches, heart palpitations, aches and joint pain, problems with memory or concentration). Thirdly, participants were asked how much of a negative impact, if any, they thought menopause had on three aspects of females’ lives: physical health, mental health and working lives (response options: 1 [no impact] to 5 [major impact], do not know). Responses were dichotomised to identify perception of a large negative impact (responses of 4 and 5 = 1, other responses = 0). Finally, participants were asked how much they strongly agreed (1) to strongly disagreed (5) with three statements: “I would feel comfortable talking about menopause with my family and friends”; “More public discussion is needed on menopause to increase public understanding”; and “Workplaces should provide support to females experiencing symptoms of the menopause”. Responses were dichotomised to identify agreement with the statements (strongly agree, agree = 1; other responses = 0).

The demographic measures included sex, age, postcode of residence, and ethnicity. Sex was asked as male, female, other, or prefer not to say. Due to low numbers, the latter two groups could not be included in this analysis. Age was collected as date of birth or year of birth and categorised into four groups (16–29, 30–49, 50–69, and 70+ years). Postcode was coded by the MRC into deprivation quintiles using the WIMD 2019. Ethnicity was collected using UK census categories. Due to low numbers in other than white ethnicity categories, binary codes were created (white [including white minorities], other than white).

Of existing panel members (n = 2526) at the time of survey launch, 32% completed the April 2023 survey. An additional 239 new people completed the questionnaire.

### 2.2. Statistical Analysis

The survey was completed by 1051 participants; however, 27 participants were excluded from the analysis due to responding ‘other’ or ‘prefer not to say’ to the sex question (n = 18), responding ‘other’ or ‘prefer not to say’ to the ethnicity question (n = 5), or responding ‘prefer not to say’ to all menopause questions (n = 4). This resulted in a final analysis sample of 1020 participants (Table S2). Sample sizes vary by question reported depending on the frequency of ‘prefer not to say’ responses. Where possible, given the topic and the stigma associated, participants who reported ‘do not know’ and ‘prefer not to say’ were included in the analysis (e.g., sufficient participants reporting). However, it was not appropriate to include ‘prefer not to say’ responses for perceived knowledge, symptom awareness, or perceived impact.

Chi-square tests were used to measure bivariate relationships between outcome measures and participant socio-demographics. Independent relationships between the outcome measures and participant demographics were assessed using binary logistic regression (enter method). Findings from the regressions are presented as adjusted odds ratios (AORs) with 95% confidence intervals (CIs) and *p*-values. Statistical analyses were conducted in SPSS v.24.

## 3. Results

The demographics of the sample and survey participation method are shown in Table S2. Over two-thirds (69.3%) of the sample were female, 50.0% were aged 16–49 years and 50.0% were aged 50+ years, and 97.3% were of white ethnicity. Across deprivation quintiles, proportions ranged from 17.6% (quintile 1—most deprived) to 21.9% (quintile 4). Over three quarters of participants (76.2%) took part online, 14.2% by telephone and 9.6% face-to-face.

### 3.1. Perceived Knowledge of Menopause

One in five participants (18.9%) reported being very knowledgeable about menopause, with 50.0% reporting being fairly knowledgeable, 21.5% being not very knowledgeable and 9.6% being not at all knowledgeable (Table 1). In bivariate analyses, reported level of knowledge was significantly associated with age (*p* < 0.001), sex (*p* < 0.001) and deprivation quintile (*p* = 0.022), but not with ethnicity (*p* = 0.747). The youngest participants (aged 16–29 years), males and participants from the most deprived quintile were most likely to report being not at all knowledgeable. In multi-variate analyses, low knowledge of menopause (being not at all or not very knowledgeable) was independently associated with age (*p* < 0.001) and sex (*p* < 0.001); however, significance in deprivation did not remain (*p* = 0.547). Adjusted odds of low knowledge increased as age reduced, with 16–29-year-olds being almost seven times more likely to report low knowledge than those aged 70+ years, and males being six times more likely than females (Table 1).

### 3.2. Awareness of Menopause Symptoms

Participants were asked if they were aware of eight menopause symptoms. The three most known symptoms were hot flushes (92.2%), changes to mood (89.7%), and difficulty sleeping (76.9%), while the three least known were heart palpitations (46.5%), aches and pains (56.8%), and headaches (57.6%; Table S3). Bivariate analysis found some significant differences in symptom awareness within each demographic (Table S3). A significantly greater proportion of females than males reported being aware of each symptom (all *p* < 0.001), whilst proportions reporting awareness of all symptoms, except changes to mood, were highest amongst 50–69-year-olds and lowest in the youngest age group for all symptoms (16–29-year-olds; all *p* < 0.001). By deprivation, significant differences were found in awareness of four symptoms (hot flushes, difficulty sleeping, changes to mood and concentration, and memory problems), with those living in the most deprived quintiles less likely to report knowing the symptoms (all *p* < 0.05). Furthermore, by ethnicity, a significant difference was found between three symptoms (hot flushes, difficulty sleeping and aches and joint pain; all *p* < 0.05), with people of white ethnicity reporting greater awareness than those of other than white ethnicity.

In multi-variate analyses, while independent relationships with sex and age remained for each symptom, deprivation and ethnicity were non-significant (all *p* > 0.05; Table 2). Females consistently reported greater awareness than males. The greatest gap in awareness between sexes was for problems with memory or concentration, with females more likely to report awareness than males. Consistently, the same symptom had the biggest gap in the age groups, where compared to 70+-year-olds, those aged 30–49 were nearly twice as likely to know the symptom, those aged 50–69 were nearly three times likely to know the symptom, and those aged 16–29 years were three times less likely to know. Variations within the age groups were also found for other symptoms. 

### 3.3. Perceived Negative Impact of Menopause on Females’ Lives

Most participants perceived menopause to have a large negative impact on females’ lives, with 76.1% providing ratings of 4 or 5 for negative impact on females’ mental health (scale: 1 = no impact to 5 = major impact) and almost 70% providing ratings of 4 or 5 for negative impact on females’ physical health and working lives (Table 3 and Table S4).

Bivariate analyses showed that perceptions of negative impact differed by age and sex (all *p* < 0.001), but no significant differences were found by deprivation or ethnicity (Table S4). Specifically, females reported greater negative impacts than males, and 30–49-year-olds reported greater negative impacts than other age groups across all three areas of females’ lives. Proportions perceiving no or low negative impact (scores of 1 or 2) were highest in 16–29-year-olds for impact on females’ physical health and working lives and in 70+-year-olds for impact on mental health (Table S4).

After controlling for confounders, significant relationships remained for sex and age (all *p* < 0.001). Males were found to be significantly less likely than females to perceive a large negative impact of menopause on females’ mental health, physical health and working lives (Table 3). Age was significantly associated with perceiving large negative impacts on females’ physical and mental health, with adjusted odds highest in 30–49-year-olds (compared with 70+-year-olds) and also elevated in 50–69-year-olds for physical health and both 50–69-year-olds and 16–29-year-olds for mental health.

### 3.4. Attitudes Towards Menopause

Seven in 10 participants (72.3%) agreed they would feel comfortable talking about menopause with their family and friends. Males were significantly less likely to agree (58.5%) than females (78.4%), and this relationship remained in multivariate analyses (males AOR 0.37; Table 4). Most participants (77.8%) agreed that more public discussion is needed on menopause to increase public understanding (Table 4 and Table S5). In bivariate analyses, agreement was associated with sex, age group and deprivation, with females (81.7% vs. 69.0% males) and 30–49-year-olds (87.9% vs. 61.2% 70+-year-olds) and residents in the second least deprived quintile (81.2% vs. 74.9% least deprived; Table 4 and Table S5) reporting the highest level of agreement. In multi-variate analyses, agreement with public discussion remained independently associated with age group and sex, with 30–49-year-olds four times more likely to agree than 70+-year-olds and males half as likely as females.

Most participants (81.8%) also agreed that workplaces should provide support to females experiencing symptoms of menopause. Again, agreement was higher in females (83.3% vs. 78.6% in males) and 30–49-year-olds (90.6% vs. 71.3% in 70+-year-olds). Associations with age remained in multivariate analyses, with 30–49-year-olds nearly four times more likely to agree than 70+-year-olds. Relationships with sex were no longer significant.

## 4. Discussion

This study sought to investigate perceived knowledge, symptom awareness, attitudes towards the impact of menopause, and perceptions of the negative impact of menopause in a national cross-sectional sample of the population in Wales and to explore differences across socio-demographic groups. Most of the existing literature in this area has focused on menopausal females (including peri- and post-menopausal), and a small proportion on partners of menopausal females. Large gaps in understanding exist at a population level, and this study contributes by investigating menopause using a national general population sample and exploring outcomes by demographics. Understanding awareness and perspectives from different population groups is important to strengthen support systems and interventions to assist females as they transition through this natural life process. A key finding of this study is the absence of any differences by deprivation for any of the outcomes, which contrasts with evidence for other health conditions showing people living in more deprived areas report less health literacy [23]. Further research is needed to verify this outcome and investigate whether there are other drivers that could influence inequalities and menopause (e.g., education and employment).

### 4.1. Perceived Knowledge of Menopause

Nearly a third of our sample (31.1%) reported having low knowledge about menopause, with significant differences in perceived knowledge by sex and age (Table 1). Adjusted odds of reporting low menopause knowledge were substantially elevated in males compared with females (AOR 6.04) and in younger age groups compared with older age groups (16–29-year-olds, AOR 6.84; 30–49-year-olds, AOR 4.79, vs. 70+-year-olds; Table 1). Previous studies have predominantly focused on females’ knowledge of menopause, often in light of their preparedness for entering this stage of life. For example, in samples of females predominantly from the UK, 61% of those aged over 40 years felt they had not been informed at all about menopause before the age of 40 [12], while 45% of those aged under 40 years felt they were not informed about menopause [13]. The latter study also found a desire among females for males to be more informed. In a student sample from the USA, knowledge about menopause was also reported to be lower in males than females [8]. Identifying opportunities to increase knowledge is important, with informational assistance as a form of social support having been found to benefit women’s experiences of menopause [5]. One early opportunity includes education on menopause in the school curriculum [24]. In a sample of peri-menopausal women, school was the most rated location (83.6%) for where they thought people should learn about menopause [12]. Currently, however, menopause does not typically feature in reproductive health lessons. A study with 16- to 18-year-olds from across England showed only 10% of participants reported having been taught about menopause in school, with students actively highlighting the topic as one that should be discussed [25]. In our study, no significant differences in perceived knowledge were found by ethnicity. Evidence elsewhere has identified knowledge about menopause to be low among both ethnic minority women and white women [26,27].

### 4.2. Symptom Awareness

Most participants in our study were aware of the ‘stereotypical’ symptoms of menopause, such as hot flushes (92.2%) and changes to mood (89.7%; Table S3). This symptom awareness is consistent with existing literature which found the same two top symptoms in a sample of UK women aged 35–60 years [28]. Less awareness was found in the current study of physical symptoms such as headaches (57.6%), aches and joint pain (56.8%), and heart palpitations (46.5%). For all symptoms, males and younger age groups showed substantially lower levels of awareness. Lower symptom recognition among males [17,18] and younger age groups (e.g., students [8]) has also been reported elsewhere. Here, the proportions reporting awareness of some symptoms (e.g., hot flushes, difficulty sleeping, changes to mood, problems with concentration and memory) were lower among people living in more deprived areas (Table S3), but relationships were not significant when other demographics were controlled for (Table 2). A review of the social determinants of health and menopause identified that people from lower socio-economic conditions experienced menopausal symptoms earlier [5]. Additionally, evidence has shown that the number of symptoms experienced by females can differ by ethnic group [29]; however, no differences in symptom awareness by ethnicity were found in the current study. Understanding knowledge gaps in different socio-economic groups is important to support work to improve females’ experience. Through understanding the breadth and variation in symptoms, people will be more equipped to respond to the needs of menopausal females in a holistic way while recognising the complex and nuanced symptom experiences of individuals.

### 4.3. Perceived Negative Impact of Menopause

Overall, most participants perceived that menopause has a large negative impact on females’ lives in terms of their mental health (76.1%), working lives (69.7%) and physical health (69.6%; Table 3). Consistent with the lower levels of symptom awareness reported by males, they were less likely than females to perceive a large negative impact of menopause on females across all three domains, including when controlling for confounding variables. The age group that perceived the largest negative impact across all domains was 30–49-year-olds, with odds in this age group being substantially elevated compared with 70+-year-olds and higher than those for the 50–69-year-olds. This may reflect increased media attention and reduced stigma about menopause over the past two decades [9], meaning younger populations are likely to have been exposed to more normalised and open communication about the impacts of menopause. While this may have increased awareness of the potential negative impacts, it may also have increased perceptions of negative impacts. No significant differences were found in multi-variate analyses between age groups for perceived negative impacts on the working lives of females. Perceptions of the level of negative impact also did not vary by ethnicity; however, where other domains of impact have been measured in menopausal females, differences in ethnicity have been found [30].

### 4.4. Attitudes Towards Menopause

Nearly three-quarters of our sample (72.3%) reported they would be comfortable talking about menopause with family and friends, rising from 58.5% of males to 78.4% of females (Table 4). Elsewhere, in an online sample of females under 40 years (predominantly from the UK), only around 40% reported that they would feel able to talk about menopause with family and friends when the topic is brought up [13]. In our study, conducted a year after [13], the proportion reporting they would be comfortable was lowest in the youngest age group (16–29 years) yet still reached 62.9% (of both sexes; Table 4). However, sampling variations may account for some of these differences. Evidence shows that females prefer to obtain information on menopause from friends [12,31]. Given the favouring of informal advice on menopause, supporting more people to feel comfortable to discuss the subject and ensuring that people of all ages are accurately informed to reduce misinformation and further negative stigmatising when having these conversations is important, particularly as poor support can increase negative experience [6]. The lack of significant differences by deprivation and ethnicity may be reflective of a general difficulty across areas and cultures to discuss the topic of menopause [9].

One approach to increase knowledge about menopause is to further increase public discussion on the topic. Three in four females in the UK have previously reported perceiving menopause to be a taboo subject which is not openly discussed [28]. It has separately been perceived as a taboo subject in the workplace [32]. From a population perspective, this study found three in four people (77.8%) agreed that more public discussion is needed on menopause to increase public understanding, with females (81.7%) and 30–49-year-olds (87.9%) most in support (Table 4). This could be connected to their increased awareness of the impact of menopause compared to other population groups. In addition, most of our sample were supportive of workplaces providing more support to females experiencing symptoms of menopause. The younger age groups (16–49 years) were more supportive than the older groups (50+ years; Table 4), which could reflect the increased focus on menopause in the workplace in the UK following recent government discussions [33]. Currently in the UK, the state retirement age is 66 years [34] and set to rise to 67 years from 2026 [35]. Thus, it is important to improve workplace environments to ensure they are supportive of the needs of menopausal and post-menopausal women to enable women to remain economically active [36]. Menopause in the workplace has become an important topic of research [37].

### 4.5. Limitations

Whilst this study sought to provide a national population perspective on perceived knowledge of and attitudes towards menopause, this study is not without its limitations. One of the study objectives was to explore perceived knowledge instead of testing actual knowledge of menopause. As this study was part of a larger population study delivered by a national public health agency, we did not want to risk the spread of misinformation on an already taboo topic by including false statements and symptoms; however, it would be beneficial for research to consider the differences in perceived and actual knowledge and awareness within different population groups using a testing approach.

From a methodological perspective, a quota sampling approach was used to obtain a nationally representative sample for the population panel accessed to complete this survey as well as for the survey sample itself. However, males were under-represented in the sample achieved. This was reflective of typical panel survey participation rates as opposed to the topic itself, with menopause being just one of the various public health topics covered in the questionnaire. Nonetheless, more work is needed to engage males and minority populations in issues around menopause and to understand their experiences, knowledge and attitudes. Due to the small number of respondents to the survey who described their sex as neither male nor female, their responses could not be included in the analysis. The menopause experiences of people who do not identify to binary gender norms or are transgender are being recognised as an important topic of study [6], but research is currently limited [38]. Additionally, due to the sampling being representative of the population of Wales and the diversity of ethnicity in Wales being limited, the results by ethnicity should be interpreted with caution. Therefore, more focused research is needed with different ethnic groups.

More research on the diverse experiences of menopause and of those in the environment to support people experiencing menopause is needed. It must also be acknowledged that individuals who participate in a public health panel without any remuneration, such as the one used for this study, may have an increased interest in public health, and therefore their responses may be subject to affinity bias. Additionally, recruitment and participation methods may have influenced participants’ responses. Due to demographically targeted approaches (e.g., targeted face-to-face interviews of 16–29-year-olds), the participation method could not be accounted for within the models.

## 5. Conclusions

This national cross-sectional study is important as it highlights socio-demographic differences in knowledge on menopause, with gaps identified specifically among males and younger age groups. Population-level insight of knowledge and perceived negative impact of menopause, along with the agreement on the need for more public awareness, can be used by researchers, practitioners, and policymakers to design public health and health education interventions to improve females’ experience of menopause and consequentially improve their health and well-being.

## Figures and Tables

**Table 1:** Perceived knowledge of menopause by participant socio-demographics and adjusted odds ratios (AORs) for low knowledge.

How Knowledgeable, If at All, Would You Say You Are About the Menopause? (%)						Low Knowledge of Menopause (Not at All or Not Very)	
		Not at All Knowledgeable	Not Very Knowledgeable	Fairly Knowledgeable	Very Knowledgeable	AOR (95% CI)	*p*
All (n = 1013) *		9.6	21.5	50.0	18.9		
Age group (years)	16–29	24.4	28.6	41.7	5.4	6.84 (3.95–11.83)	<0.001
	30–49	7.1	29.6	53.0	10.4	4.79 (2.90–7.89)	<0.001
	50–69	6.9	14.2	48.6	30.2	1.42 (0.86–2.33)	0.173
	70+	5.1	13.1	55.1	26.7	REF	<0.001
	X2				136.514		
	*p*				<0.001		
Sex	Female	3.7	17.7	52.7	25.9	REF	
	Male	23.1	30.3	44.0	2.6	6.04 (4.33–8.43)	<0.001
	X2				165.167		
	*p*				<0.001		
Deprivation quintile	1—Most	17.5	22.0	45.8	14.7	1.48 (0.91–2.40)	0.112
	2	7.4	25.4	46.6	20.6	1.41 (0.87–2.27)	0.162
	3	9.1	20.2	51.9	18.8	1.32 (0.83–2.12)	0.245
	4	7.7	22.1	48.2	22.1	1.30 (0.82–2.06)	0.264
	5—Least	7.4	18.4	56.7	17.5	REF	0.547
	X2				23.764		
	*p*				0.022		
Ethnicity	White ^	9.4	21.4	50.2	19.0	REF	
	Other than white	14.3	25.0	46.4	14.3	0.88 (0.37–2.10)	0.768
	X2				1.223		
	*p*				0.747		

* Excludes seven participants who responded ‘prefer not to say’ to the question. ^ White (including white minority). REF = reference category. *p*-values in the REF rows relate to the overall impact made by each independent variable on the model.

**Table 2:** Adjusted odds ratios (AORs) for reporting knowing symptoms of menopause by participant socio-demographics.

		Hot Flushes		Difficulty Sleeping		Weight Gain		Mood Changes		Headaches		Heart Palpitations		Aches and Joint Pain		Problems with Memory or Concentration	
All (N = 1016) *		AOR (95% CI)	*p*	AOR (95% CI)	*p*	AOR (95% CI)	*p*	AOR (95% CI)	*p*	AOR (95% CI)	*p*	AOR (95% CI)	*p*	AOR (95% CI)	*p*	AOR (95% CI)	*p*
Age group (years)	16–29	0.20 (0.09–0.42)	<0.001	0.16 (0.09–0.27)	<0.001	0.42 (0.26–0.68)	<0.001	0.22 (0.12–0.42)	<0.001	0.57 (0.37–0.89)	0.014	0.43 (0.26–0.69)	0.001	0.60 (0.38–0.94)	0.024	0.31 (0.19–0.51)	<0.001
	30–49	0.94 (0.40–2.16)	0.877	0.80 (0.48–1.33)	0.386	1.25 (0.81–1.93)	0.304	1.64 (0.78–3.48)	0.195	1.15 (0.79–1.69)	0.463	1.33 (0.90–1.95)	0.150	1.33 (0.91–1.94)	0.147	1.86 (1.17–2.95)	0.008
	50–69	1.65 (0.70–3.93)	0.256	1.56 (0.93–2.61)	0.093	2.09 (1.34–3.24)	0.001	1.68 (0.83–3.42)	0.148	1.39 (0.95–2.04)	0.088	1.95 (1.33–2.86)	0.001	1.84 (1.25–2.69)	0.002	2.75 (1.73–4.37)	<0.001
	70+	REF	<0.001		<0.001		<0.001		<0.001		<0.001		<0.001		<0.001		<0.001
Sex	Female	REF															
	Male	0.14 (0.08–0.24)	<0.001	0.17 (0.12–0.25)	<0.001	0.25 (0.18–0.34)	<0.001	0.19 (0.12–0.30)	<0.001	0.52 (0.39–0.69)	<0.001	0.43 (0.32–0.58)	<0.001	0.45 (0.34–0.60)	<0.001	0.15 (0.11–0.22)	<0.001
Deprivation quintile	1—Most	0.76 (0.36–1.63)	0.483	0.86 (0.51–1.47)	0.589	0.92 (0.57–1.48)	0.739	0.62 (0.31–1.24)	0.175	0.84 (0.55–1.27)	0.406	1.18 (0.77–1.82)	0.446	0.96 (0.63–1.46)	0.845	0.63 (0.38–1.04)	0.071
	2	0.94 (0.41–2.13)	0.881	0.85 (0.50–1.45)	0.561	0.97 (0.60–1.56)	0.900	0.71 (0.34–1.50)	0.371	1.03 (0.69–1.56)	0.872	1.13 (0.75–1.71)	0.548	1.26 (0.83–1.90)	0.273	0.86 (0.51–1.43)	0.555
	3	1.21 (0.53–2.77)	0.646	0.99 (0.57–1.66)	0.957	0.92 (0.58–1.44)	0.703	1.05 (0.50–2.23)	0.893	1.12 (0.75–1.67)	0.568	1.25 (0.84–1.87)	0.270	1.09 (0.73–1.62)	0.675	0.98 (0.60–1.62)	0.951
	4	1.55 (0.66–3.65)	0.315	1.06 (0.63–1.78)	0.821	1.22 (0.77–1.93)	0.398	1.01 (0.48–2.11)	0.989	1.01 (0.69–1.49)	0.958	1.28 (0.87–1.90)	0.213	1.28 (0.86–1.90)	0.221	1.16 (0.71–1.91)	0.559
	5—Least	REF	0.506		0.914		0.756		0.446		0.745		0.754		0.551		0.181
Ethnicity	White ^	REF															
	Other than white	0.70 (0.22–2.28)	0.559	0.77 (0.31–1.96)	0.590	1.10 (0.46–2.67)	0.825	1.03 (0.32–3.29)	0.960	0.78 (0.36–1.72)	0.545	0.87 (0.38–2.03)	0.752	0.51 (0.23–1.17)	0.112	0.79 (0.31–2.04)	0.632

* Excludes eight participants who responded ‘prefer not to say’ to the question set. ^ White (including white minority). REF = reference category. *p*-values in the REF rows relate to the overall impact made by each independent variable on the model.

**Table 3:** Proportions and adjusted odds ratios (AORs) for perceived large negative impact of menopause on females’ lives by participant socio-demographics.

		Physical Health				Mental Health			Working Lives	
		%	AOR (95% CI)	*p*	%	AOR (95% CI)	*p*	%	AOR (95% CI)	*p*
All (n = 1013) *		69.6			76.1			69.7		
Age group (years)	16–29	54.4	0.73 (0.47–1.15)	0.176	64.5	1.06 (0.67–1.69)	0.791	54.4	0.75 (0.48–1.17)	0.202
	30–49	79.4	2.22 (1.47–3.36)	<0.001	90.0	4.70 (2.90–7.60)	<0.001	79.9	2.35 (1.55–3.55)	<0.001
	50–69	72.7	1.71 (1.15–2.54)	0.008	75.2	1.77 (1.18–2.65)	0.006	72.7	1.74 (1.17–2.59)	0.006
	70+	59.4	REF	<0.001	62.3		<0.001	58.9		<0.001
Sex	Female	75.3	REF		82.1			75.0		
	Male	56.5	0.48 (0.36–0.64)	<0.001	62.3	0.43 (0.31–0.59)	<0.001	57.5	0.51 (0.38–0.68)	<0.001
Deprivation quintile	1—Most	69.1	1.45 (0.92–2.78)	0.106	74.2	1.37 (0.85–2.23)	0.199	67.4	1.43 (0.92–2.24)	0.113
	2	71.8	1.25 (0.81–1.94)	0.318	77.1	1.20 (0.75–1.94)	0.446	75.0	1.60 (1.03–2.50)	0.038
	3	72.5	1.44 (0.93–2.20)	0.099	76.8	1.37 (0.87–2.18)	0.178	72.5	1.57 (1.02–2.0)	0.039
	4	69.5	1.18 (0.78–1.79)	0.427	80.7	1.68 (1.05–2.69)	0.029	70.4	1.36 (0.90–2.05)	0.148
	5—Least	65.4	REF	0.438	71.4		0.272	63.6		0.189
Ethnicity	White ^	69.6	REF		76.2			69.5		
	Other than white	67.9	1.08 (0.46–2.54)	0.858	71.4	0.84 (0.34–2.06)	0.697	75.0	1.58 (0.64–3.95)	0.324

* Excludes 11 participants who responded ‘prefer not to say’ to the question set. ^ White (including white minority). REF = reference category. *p*-values in the REF rows relate to the overall impact made by each independent variable on the model.

**Table 4:** Proportions and adjusted odds ratios (AORs) for agreement (strongly agree/agree) with attitudinal statements towards menopause by participant socio-demographics.

		I Would Feel Comfortable Talking About Menopause with My Family and Friends			More Public Discussion Is Needed on the Menopause to Increase Public Understanding			Workplaces Should Provide Support to Women Experiencing Symptoms of the Menopause		
		%	AOR (95% CI)	*p*	%	AOR (95% CI)	*p*	%	AOR (95% CI)	*p*
All (n = 1018) *		72.3			77.8			81.8		
Age group (years)	16–29	62.9	0.56 (0.34–0.91)	0.018	78.8	2.45 (1.49–4.02)	<0.001	82.9	2.02 (1.19–3.43)	0.010
	30–49	73.8	0.76 (0.49–1.18)	0.217	87.9	4.28 (2.71–6.74)	<0.001	90.6	3.80 (2.31–6.26)	<0.001
	50–69	74.2	0.90 (0.58–1.38)	0.624	75.8	1.98 (1.33–2.96)	0.001	77.9	1.45 (0.95–2.20)	0.086
	70+	74.7	REF	0.073	61.2		<0.001	71.3		<0.001
Sex	Female	78.4	REF		81.7			83.3		
	Male	58.5	0.37 (0.28–0.50)	<0.001	69.0	0.59 (0.43–0.81)	0.001	78.6	0.88 (0.62–1.24)	0.463
Deprivation quintile	1—Most	71.1	1.05 (0.66–1.67)	0.832	75.6	0.96 (0.59–1.56)	0.872	80.0	0.90 (0.54–1.50)	0.679
	2	70.9	0.88 (0.56–1.37)	0.562	76.7	0.93 (0.58–1.50)	0.780	81.5	0.94 (0.57–1.57)	0.821
	3	71.0	0.85 (0.55–1.32)	0.469	80.2	1.32 (0.82–2.12)	0.251	81.2	1.04 (0.63–1.70)	0.881
	4	74.4	1.00 (0.65–1.55)	0.989	81.2	1.40 (0.87–2.24)	0.163	85.7	1.41 (0.84–2.34)	0.191
	5—Least	73.5	REF	0.862	74.9		0.339	80.4		0.515
Ethnicity	White ^	72.8	REF		77.7			81.6		
	Other than white	53.6	0.49 (0.22–1.07)	0.073	82.1	1.19 (0.43–3.29)	0.742	89.3	1.65 (0.48–5.65)	0.426

* Excludes two participants who did not answer the question set due to a technical system error. ^ White (including white minority). REF = Reference category. *p*-values in the REF rows relate to the overall impact made by each independent variable on the model.

## Data Availability

The datasets used and analysed during the current study are available from the project team on reasonable request (talkphwales@wales.nhs.uk).

## Supplementary Materials

The following supporting information can be downloaded at: https://www.mdpi.com/article/10.3390/ijerph22020287/s1. Table S1: Variables included in the analysis. Table S2: Demographics of the survey sample. Table S3: Knowledge of menopause symptoms by participant socio-demographics. Table S4: Perception of the negative impact of menopause on females’ lives by participant socio-demographics. Table S5: Attitudinal perspectives towards menopausal-related statements by participant demographics.
