# Supplementary material for: Where Are the Knowledge Gaps in Menopause Across a Population? A National Cross-Sectional Survey in Wales
Source: Int J Environ Res Public Health. 2025 Feb 14;22(2):287. doi: 10.3390/ijerph22020287 (PMC11855723; doi:10.3390/ijerph22020287)
Supplement: Supplementary file 1 [file ijerph-22-00287-s001.zip › ijerph-3289793-supplementary.pdf]

Supplementary Material

Table S1. Variables included in the analysis.

	Question (Response Options)	Qualifying Response
Knowledge of menopause	How knowledgeable, if at all, would you say you are about the menopause? (Not at all knowledgeable; Not very knowledgeable; Fairly knowledgeable; Very knowledgeable; Prefer not to say)	Not at all; Not very
Awareness of menopausal symptoms	Women can experience a range of symptoms during the menopause, and we are interested in learning whether people in Wales recognize these. Which of the following did you already know could be a symptom? (Yes)	Yes
	Hot flushes	
	Difficulty sleeping	
	Weight gain	
	Changes to mood (e.g., anxiety, mood swings)	
	Headaches	
	Heart palpitations	
	Aches and joint pain	
	Problems with memory or concentration	
	Don't know	
	Prefer not to say	
Perceived impact of menopause on a women's life	How much of a negative impact, if any, do you think menopause has on the following aspects of women's lives? (1—No impact at all, 5—Major impact; Don't know; Prefer not to say)	4–5
	Women's physical health	
	Women's mental health	
	Women's working lives	
Attitudes towards menopause	To what extent do you agree or disagree with the following statements: (Strongly disagree; Disagree; Neither agree nor disagree; Agree; Strongly Agree; Prefer not to say)	Strongly agree; Agree
	I would feel comfortable talking about menopause with my family and friends	
	More public discussion is needed on the menopause to increase public understanding	
	Workplaces should provide support to women experiencing symptoms of the menopause	

Table S2. Demographics of the survey sample.

All	n	%
	1020	100
Age group (years)		
16–29	170	16.7
30–49	340	33.3
50–69	332	32.5
70+	178	17.5
Sex		
Female	707	69.3
Male	313	30.7
Deprivation quintile		
1—Most	180	17.6
2	189	18.5
3	209	20.5
4	223	21.9
5—Least	219	21.5
Ethnicity		
White (including ethnic minority white)	992	97.3
Other than white	28	2.7
Participation method		
Online	777	76.2
Telephone	145	14.2
Face-to-face	98	9.6

Table S3. Knowledge of menopause symptoms by participant socio-demographics.

Women Can Experience a Wide Range of Symptoms when Going Through the Menopause. Which of the Following Did You Already Know Could Be a Symptom? Yes (%)										
		**Hot Flushes**	**Difficulty Sleeping**	**Weight Gain**	**Changes to Mood**	**Headaches**	**Heart Palpitations**	**Aches & Joint Pain**	**Conc & Memory Problems ~**	**Don't Know**
All (N = 1012) *		92.2	76.9	71.4	89.7	57.6	46.5	56.8	72.1	4.9
Age group (years)	16–29	75.9	44.1	47.6	68.8	40.6	22.9	37.1	39.4	17.1
	30–49	95.6	82.5	76.9	95.6	61.5	51.2	61.2	82.2	1.2
	50–69	96.4	86.6	80.9	94.2	63.8	57.4	66.0	82.7	2.7
	70+	93.7	79.4	66.3	90.3	54.9	40.0	50.3	64.6	4.6
	X2	76.644	126.972	68.712	100.321	28.060	59.719	43.993	130.925	66.771
	p	<0.001	<0.001	<0.001	<0.001	<0.001	<0.001	<0.001	<0.001	<0.001
Sex	Female	96.9	86.0	80.3	95.0	62.8	52.9	62.9	83.5	1.1
	Male	81.3	55.7	50.8	77.4	45.6	31.8	42.6	45.9	13.8
	X2	71.837	109.756	91.010	72.173	25.893	38.115	35.856	149.464	72.473
	p	<0.001	<0.001	<0.001	<0.001	<0.001	<0.001	<0.001	<0.001	<0.001
Deprivation quintile	1—Most	85.3	67.8	64.4	81.4	49.7	41.8	49.2	58.8	9.6
	2	92.6	77.1	72.9	89.9	59.6	47.9	61.2	73.9	5.9
	3	93.8	78.5	70.8	91.9	60.8	48.8	56.9	74.2	3.8
	4	95.1	80.3	75.8	91.9	58.7	49.8	61.0	77.1	2.7
	5—Least	93.0	79.1	72.1	92.1	58.1	43.7	54.9	74.4	3.7
	X2	15.180	10.540	6.629	16.972	5.806	3.783	7.599	19.816	12.165
	p	0.004	0.032	0.157	0.002	0.214	0.436	0.107	0.001	0.016
Ethnicity	White ^	92.5	77.3	71.6	89.9	57.9	46.8	57.4	72.6	4.9
	Other than white	82.1	60.7	64.3	82.1	46.4	35.7	35.7	57.1	7.1
	X2	4.042	4.232	0.723	1.795	1.474	1.357	5.227	3.220	0.297
	p	0.044	0.040	0.395	0.180	0.255	0.244	0.022	0.073	0.586

* Excludes eight participants who responded 'Prefer not to say' to the question set; ^ White (including white minority); ~ Problems with memory or concentration.

Table S4. Perception of the negative impact of menopause on females' lives by participant socio-demographics.

	Physical Health (%)					Mental Health (%)					Working Lives (%)				
	**No/Low Impact (1&2)**	**3**	**4**	**5—Major Impact**	**Don't Know**	**No/Low Impact (1&2)**	**3**	**4**	**5—Major Impact**	**Don't Know**	**No/Low Impact (1&2)**	**3**	**4**	**5—Major Impact**	**Don't Know**
All (N = 1013) *	5.2	18.2	33.1	36.5	7.0	3.9	13.4	30.3	45.8	6.5	6.2	16.9	32.8	36.9	7.2
Age group (years)															
16–29	11.2	20.1	34.9	19.5	14.2	5.3	17.8	25.4	39.1	12.4	16.6	13.6	31.4	23.1	15.4
30–49	1.2	14.2	33.6	45.7	5.3	0.3	6.8	33.0	56.9	2.9	1.5	14.7	37.8	42.2	3.8
50–69	4.8	17.3	32.4	40.3	5.2	3.6	15.2	29.1	46.1	6.1	3.6	17.6	30.6	42.1	6.1
70+	8.0	25.7	31.4	28.0	6.9	10.3	18.9	32.0	30.3	8.6	10.3	22.9	28.6	30.3	8.0
X2					75.831					87.669					97.041
p					<0.001					<0.001					<0.001
Sex															
Female	4.1	17.3	32.5	42.8	3.3	3.0	12.8	27.8	54.3	2.1	4.5	17.0	32.5	42.6	3.4
Male	7.8	20.1	34.4	22.1	15.6	6.2	14.9	36.0	26.3	16.6	10.1	16.6	33.4	24.0	15.9
X2					78.453					116.350					77.054
p					<0.001					<0.001					<0.001
Deprivation quintile															
1—Most	5.1	16.9	36.5	32.6	9.0	4.5	12.4	30.9	43.3	9.0	5.6	16.3	32.6	34.8	10.7
2	5.3	15.4	33.5	38.3	7.4	3.7	13.3	27.7	49.5	5.9	4.8	13.8	34.0	41.0	6.4
3	3.9	16.4	32.9	39.6	7.2	3.9	12.6	26.6	50.2	6.8	6.3	12.6	34.3	38.2	8.7
4	7.2	18.8	28.3	41.3	4.5	4.5	10.3	33.6	47.1	4.5	8.5	17.0	29.1	41.3	4.0
5—Least	4.6	22.6	35.0	30.4	7.4	3.2	18.4	32.3	39.2	6.9	5.5	24.0	34.1	29.5	6.9
X2					16.844					16.177					26.280
p					0.396					0.441					0.050
Ethnicity															
White ^	5.3	18.3	33.2	36.4	6.8	4.1	13.4	30.4	45.9	6.3	6.4	17.1	32.4	37.2	7.0
Other than white	3.6	14.3	28.6	39.3	14.3	0.0	14.3	28.6	42.9	14.3	0.0	10.7	46.4	28.6	14.3
X2					2.802					3.905					6.624
p					0.591					0.419					0.157

* Excludes 11 participants who responded 'Prefer not to say' to the question set. ^ White (including white minority).

Table S5. Attitudinal perspectives towards menopausal-related statements by participant demographics.

	I Would Feel Comfortable Talking About Menopause with My Family and Friends (%)				More Public Discussion is Needed on the Menopause to Increase Public Understanding (%)				Workplaces Should Provide Support to Women Experiencing Symptoms of the Menopause (%)			
	Strongly Agree/Agree	Neither	Strongly Disagree/Disagree	Prefer Not to Say	Strongly Agree/Agree	Neither	Strongly Disagree/Disagree	Prefer Not to Say	Strongly Agree/Agree	Neither	Strongly Disagree/Disagree	Prefer Not to Say
All (N = 1018) *	72.3	15.0	9.7	2.9	77.8	12.7	5.9	3.6	81.8	11.3	4.3	2.6
Age group (years)												
16–29	62.9	15.3	12.9	8.8	78.8	5.3	1.2	14.7	82.9	5.9	1.8	9.4
30–49	73.8	16.8	8.8	0.6	87.9	8.5	2.4	1.2	90.6	7.4	1.5	0.6
50–69	74.2	12.1	11.5	2.1	75.8	14.2	8.5	1.5	77.9	14.5	6.4	1.2
70+	74.7	16.9	5.1	3.4	61.2	24.7	12.4	1.7	71.3	18.0	8.4	2.2
X2				39.807				142.866				83.371
p				<0.001				<0.001				<0.001
Sex												
Female	78.4	13.8	7.4	0.4	81.7	10.5	6.5	1.3	83.3	11.2	4.7	0.9
Male	58.5	17.9	15.0	8.6	69.0	17.6	4.5	8.9	78.6	11.5	3.5	6.4
X2				76.901				49.677				27.313
p				<0.001				<0.001				<0.001
Deprivation quintile												
1—Most	71.1	12.2	10.6	6.1	75.6	9.4	6.1	8.9	80.0	8.9	5.6	5.6
2	70.9	14.8	11.1	3.2	76.7	13.8	4.2	5.3	81.5	11.1	4.8	2.6
3	71.0	15.0	12.1	1.9	80.2	11.1	6.8	1.9	81.2	12.1	4.8	1.9
4	74.4	16.6	7.6	1.3	81.2	12.6	4.9	1.3	85.7	10.3	2.7	1.3
5—Least	73.5	16.0	7.8	2.7	74.9	16.0	7.3	1.8	80.4	13.7	4.1	1.8
X2				14.168				29.000				13.530
p				0.290				0.004				0.332
Ethnicity												
White ^	72.8	14.7	9.6	2.8	77.7	13.0	5.9	3.4	81.6	11.6	4.3	2.4
Other than white	53.6	25.0	14.3	7.1	82.1	0.0	7.1	10.7	89.3	0.0	3.6	7.1
X2				5.637				7.765				5.860
p				0.131				0.051				0.119

* Excludes two participants who did not answer the question set due to a technical system error. ^ White (including white minority).
